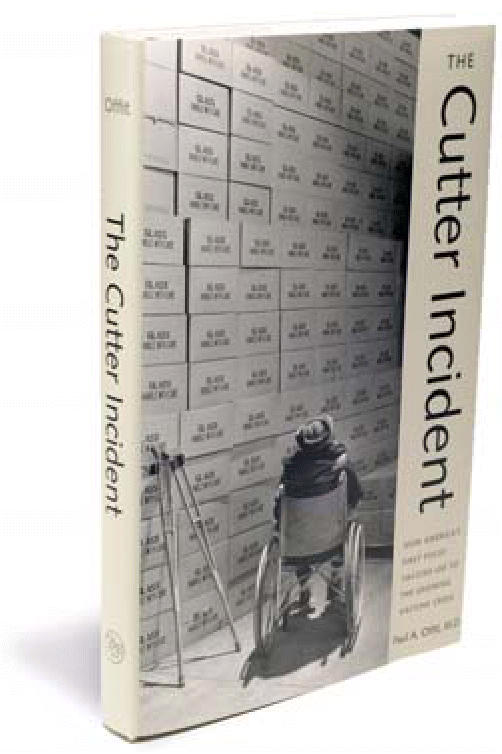# The Cutter Incident: How America’s First Polio Vaccine Led to the Growing Vaccine Crisis

**Published:** 2006-09

**Authors:** John Treanor

**Affiliations:** John Treanor is a professor of medicine in the Infectious Diseases Division at the University of Rochester Medical Center. His research interests center on the clinical development of new vaccines, particularly for influenza and other respiratory viruses

By Paul A. Offit

New Haven:Yale University Press, 2005. 238 pp. ISBN: 0-300-10864-8, $27.50

This entertaining, well-written book provides valuable insights into three interrelated areas, all built around the story of the Cutter incident, a landmark case that in some ways continues to have implications for product liability and the health of the vaccine industry today. The author, Paul Offit, has an admirable ability to present complicated issues in a way that is easy to understand for both technical and nontechnical audiences, and the book should (and does) have wide appeal.

The first part of the story deals with the actual incident, which involved the failure to completely inactivate the very first lots of inactivated poliovaccine licensed for use in the United States, and the resulting epidemic of iatrogenic paralytic polio. This incident holds many lessons for us today. Offit shows how the ultimate outbreak was really the consequence of multiple factors: an inactivation procedure that was not completely understood by the manufacturers, testing algorhythms that were relatively insensitive for detection of remaining live virus in the final preparations, the use of an extremely virulent virus as the starting material for one of the three strains, and, most unpredictable of all, the enormous consequences of a seemingly trivial process change—the substitution of glass filters in a critical step in the processing of the culture lysates. Most disasters are the result of multiple synergistic failures, not all of which are predictable, and the Cutter incident is a good illustration of this.

The second part of the story describes the resulting product liability trial, in which the manufacturer of the vaccine, Cutter Laboratories, was found to be liable even though it was not negligent. Cutter followed all available guidelines in production of the vaccine and only released lots that had passed recommended safety tests. However, the vaccine, rather than preventing polio as it was supposed to do, actually caused polio instead. In the trial, the jury did not find Cutter to have been negligent in the manufacture of the vaccine. However, Cutter was found to have violated the implied warranty that its product, when used as directed, would be safe and effective for its intended purpose. The author makes a convincing argument that this case set a precedent for liability without negligence that ultimately almost destroyed the vaccine industry in the United States. Only the Vaccine Injury Compensation Program, in which claims against manufacturers are first arbitrated by a panel of experts, has kept manufacturers in the game.

The third part of the story is a more speculative discussion of the role product liability has played in the currently tenuous situation facing our supply of critical vaccines in the United States. This complex situation involves not only product liability but the economics of the marketplace, the role of government funding for vaccines, and probably other issues as well. However, product liability clearly also plays a role, and continues to have an impact today. For example, legislation protecting manufacturers of potential pandemic vaccines against product liability claims has emerged as a critical issue, which will be required for adequate control of the current avian influenza threat, much as it was in 1976. Thimerosal, which has now been convincingly demonstrated to have no association with neurodevelopmental disorders, has in the meantime continued to be a source of liability claims and state legislation.

Vaccines have had a profound influence on human health and have been responsible for the complete elimination of smallpox, the elimination of measles and polio transmission in the Western Hemisphere, and many other benefits. However, to be effective, vaccines must be administered to very large numbers of healthy individuals. For this reason, vaccine safety always has been, and will remain, a preeminent concern among those involved in controlling human disease. This book should be of value to anyone who must deal with issues related to exposure of large populations to interventions.

## Figures and Tables

**Figure:**